# Lynch syndrome pre-screening and comprehensive characterization in a multi-center large cohort of Chinese patients with colorectal cancer

**DOI:** 10.20892/j.issn.2095-3941.2021.0585

**Published:** 2022-06-01

**Authors:** Yan Li, Lihong Fan, Jianming Zheng, Xiu Nie, Yu Sun, Qin Feng, Shenyi Lian, Wenqi Bai, Weijing Cai, Yanan Yang, Bo Su, Yanfeng Xi, Dongmei Lin

**Affiliations:** 1 Department of Pathology, Union Hospital, Tongji Medical College, Huazhong University of Science and Technology, Wuhan 430022, China; 2 Department of Respiration Medicine, Shanghai Tenth People’s Hospital, School of Medicine, Tongji University, Shanghai 200072, China; 3 Department of Pathology, Changhai Hospital of Shanghai, Shanghai 200433, China; 4 Department of Pathology, Key Laboratory of Carcinogenesis and Translational Research (Ministry of Education), Peking University Cancer Hospital & Institute, Beijing 100142, China; 5 Department of Colorectal Surgery, Shanxi Cancer Hospital, Taiyuan 030013, China; 6 Shanghai Tongshu Biotechnology Co., Ltd, Shanghai 200120, China; 7 Department of Biochemistry and Molecular Biology, Mayo Clinic, Rochester, MN 55905, USA; 8 Central Laboratory, Shanghai Pulmonary Hospital, School of Medicine, Tongji University, Shanghai 200433, China; 9 Department of Pathology, Shanxi Cancer Hospital, Taiyuan 030013, China

**Keywords:** Microsatellite instability, mismatch repair, sequencing, Lynch syndrome, somatic genetic characteristics

## Abstract

**Objective:**

Lynch syndrome (LS) pre-screening methods remain under-investigated in colorectal cancers (CRCs) in Asia. Here, we aimed to systematically investigate LS pre-screening and comprehensively characterize LS CRCs.

**Methods:**

Microsatellite instability (MSI) and germline variants of DNA mismatch repair (MMR) genes were examined in 406 deficient MMR (dMMR) and 250 proficient MMR CRCs. The genetic differences between LS and sporadic CRCs were studied with whole exome sequencing analysis.

**Results:**

The incidence of dMMR in Chinese patients with CRCs was 13.8%. Consistency analysis between MMR immunohistochemistry (IHC) and MSI testing showed the kappa value was 0.758. With next-generation sequencing (NGS), germline variants were detected in 154 CRCs. Finally, 88 patients with CRC were identified as having LS by Sanger sequencing. Among them, we discovered 21 previously unreported pathogenic germline variants of MMR genes. Chinese patients with LS, compared with sporadic CRCs, tended to be early-onset, right-sided, early-stage and mucinous. Overall, the performance of MMR IHC and MSI testing for LS pre-screening was comparable: the area under the ROC curve for dMMR, MSI-H, and MSI-H/L was 0.725, 0.750, and 0.745, respectively. dMMR_MSI-H LS and sporadic CRCs showed substantial differences in somatic genetic characteristics, including different variant frequencies of *APC*, *CREBBP*, and *KRAS*, as well as different enriched pathways of VEGF, Notch, TGFβR, mTOR, ErbB, and Rac protein signal transduction.

**Conclusions:**

MMR IHC and MSI testing were effective methods for LS pre-screening. The revealed clinical and somatic genetic characteristics in LS CRCs may have the potential to improve the performance of LS pre-screening in combination with dMMR/MSI.

## Introduction

Lynch syndrome (LS) is the most frequent hereditary colorectal cancer (CRC) syndrome^1^. It originates from germline defects in DNA mismatch repair (MMR) genes (*MLH1*, *MSH2*, *MSH6*, *PMS2*, and *EPCAM*)^2^. It is clinically characterized by an elevated risk of diverse cancers, which may occur synchronously or metachronously with relative early-onset in people with family members with LS^3^. Patients with LS and their high-risk relatives can benefit from intensive cancer surveillance, chemoprevention^4^, and risk-reducing surgeries^5^, particularly when they are identified sufficiently early.

Universal molecular screening for LS in newly diagnosed CRCs is routinely recommended by the NCCN Guidelines^2^. Paired tumor/germline or tumor multi-gene panel next-generation sequencing (NGS) has recently been proposed as a method for LS screening^6,7^ providing an alternative to the traditional complex screening strategy. Given the 1%–5% incidence of LS in CRCs^8^, pre-screening with immunohistochemistry for mismatch-repair proteins (MMR IHC) or microsatellite instability (MSI) testing before multi-gene panel NGS can significantly decrease the economic burden, particularly in underdeveloped regions^9^. However, only 20%–30% of deficient MMR (dMMR) or MSI-high (MSI-H) CRCs are LS^10,11^. In addition, several molecular characteristics have been suggested to differ between sporadic and LS CRCs^12^, such as the mutation frequency of BRAF V600E^13^. To improve the efficiency of MMR IHC or MSI testing in LS pre-screening, we aimed to explore the molecular characteristics that distinguish LS from sporadic CRCs.

Several studies have been conducted to compare the performance of MMR IHC and MSI testing in LS pre-screening^10,14^, but no consensus has been reached^15,16^. Moreover, the MMR gene variant profile of LS in the Chinese population remains under-investigated.

Chinese patients with CRC were enrolled in a large multi-center cohort to investigate the consistency of MMR IHC and MSI testing, and compare their performance in LS pre-screening. To improve the efficiency of MMR IHC or MSI testing in LS pre-screening, we aimed to identify clinical and molecular characteristics distinguishing dMMR and MSI-H (dMMR_MSI-H) LS from dMMR_MSI-H sporadic CRCs. Whole exome sequencing (WES) was performed on patients with dMMR/MSI-H LS and dMMR/MSI-H sporadic CRCs. Then the clinical and molecular characteristics in each group, the MMR gene variant profile of LS, and the performance of MMR IHC and MSI testing in LS pre-screening in the Chinese population were explored. Our investigation reveals clinical and molecular characteristics that may potentially be used to improve the performance of LS pre-screening in combination with dMMR/MSI. In addition, the results reveal the potential mechanism of carcinogenesis of LS and sporadic CRCs, and may aid in the establishment of therapeutic strategies for patients with CRC.

## Materials and methods

### Patients and samples

**Figure 1:**
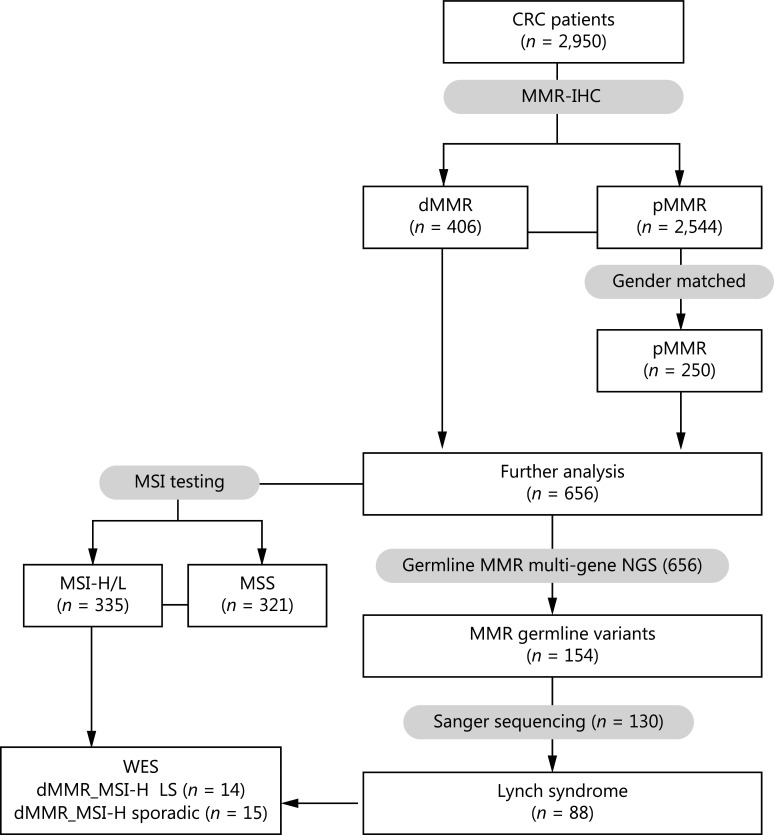
Flowchart of this research. Immunohistochemistry (IHC) staining of MMR was performed by each center, and the results were collected. MSI testing, next-generation sequencing (NGS), and Sanger sequencing were conducted by Tongshu BioTech Co., Ltd. CRC, colorectal cancer; MMR, mismatch repair; dMMR, deficient MMR; pMMR, proficient MMR; MSI, microsatellite instability; MSI-H, MSI-high; MSI-L, MSI-low; MSS, microsatellite stability.

A total of 2,950 patients with CRC were reviewed who were treated from January 2014 to December 2016 in 5 medical centers: Union Hospital, Tongji Medical College, Huazhong University of Science and Technology, located in Central China; Shanxi Cancer Hospital located in West China; Changhai Hospital of Shanghai and Shanghai Tenth People’s Hospital located in East China; and Beijing Cancer Hospital located in North China. All patients were initially screened with MMR IHC (MHL1, MSH2, MSH6, and PSM2). A cohort of 406 patients with dMMR and 250 sex-matched patients with pMMR underwent germline multi-gene panel NGS. The germline variants detected by NGS were validated by Sanger sequencing. The demographic and clinical characteristics of these 656 patients were collected. This study was approved by the ethics committees of the corresponding hospitals (approval No. 201902; [2019]Lunshenzi (5)-1; 2018TW05-ZY01), and was performed in compliance with the Declaration of Helsinki. Written informed consent was obtained from all study participants. The flowchart of this research is shown in **Figure 1**.

### MMR IHC and MSI testing

Immunohistochemistry of MLH1, MSH2, MSH6, and PMS2 for each CRC surgical specimen was routinely conducted with the corresponding monoclonal antibodies (MLH1 mouse monoclonal antibody [Mab]: ES05, MAB-0789; MSH2 rabbit MAb: RED2, RMA-0776; MSH6 rabbit MAb: EP49, RMA-0770; PMS2 rabbit MAb: EP51, RMA-0775; and MXB® Fuzhou, China). Tumors with deficient expression of one or more MMR proteins were defined as dMMR, whereas tumors with normal positive staining of all 4 proteins were defined as pMMR^17^.

MSI testing was performed with capillary electrophoresis with an NCI MSI panel kit (Tongshu BioTech., Shanghai, China). This panel contains 2 mononucleotide loci (BAT25 and BAT26), 3 dinucleotide loci (D2S123, D5S346, and D17S250), and one pentanucleotide repeat marker (Penta C) as the internal control. GeneScan Analysis and Genotyper Software packages (Applied Biosystems, CA, USA) were used to determine the predominant allele size for each locus. Finally, the MSI phenotype was determined according to the number of allelic bases and the internal control index.

### Multi-gene panel NGS

Multi-gene panel NGS, including *MLH1*, *MSH2*, *MSH6*, *PMS2*, and *EPCAM*, was performed with an Ion Torrent instrument (Tongshu BioTech, Shanghai, China). The blood samples were used for germline variant detection, and normal tissue samples were used if blood samples were unavailable. The normal tissue slides were examined by pathologists to ensure the absence of tumor cells. DNA was extracted with a DNA Extraction Kit from blood samples (150170, Changzhou Tongshu Biotechnology Co., Ltd, China) and FFPE sections (FD-50, Changzhou Tongshu Biotechnology Co., Ltd, China) according to the manufacturer’s protocols. The targeted libraries were constructed with a NGS Fast DNA Library Prep Set (Thermo Fisher). Sequencing data were analyzed in TorrentSuite-5.6 (3730XL, Life technologies) with mutation calling with VarDict.

### Sanger sequencing and identification of LS

To confirm the germline variants in MMR genes and validate the NGS results, we performed Sanger sequencing on 130 cases with MMR gene variants detected by NGS. Briefly, DNA was amplified, and PCR products were subjected to electrophoresis. The sequencing was conducted on an ABI 3730 genetic Analyzer (Applied Biosystems, CA, USA) according to the manufacturer’s protocols.

The detected variants were annotated by Clinvar. Variants not annotated by Clinvar were classified according to the American College of Medical Genetics and Genomics guidelines.

### Whole exome sequencing

DNA extracted from the normal and tumor tissue samples was isolated with a DNA Extraction Kit (FD-50, Changzhou Tongshu Biotechnology Co., Ltd, China). We created targeted capture pulldown and exon-wide libraries from native DNA with an xGen^®^ Exome Research Panel (Integrated DNA Technologies, Inc., IL, USA) and TruePrep DNA Library Prep Kit V2 for Illumina (#TD501, Vazyme, Nanjing, China), and generated paired-end sequence data with Illumina HiSeq machines with an average sequencing depth of 170× for controls and 240× for tumors. The sequence data were aligned to the human reference genome (NCBI build 37) with BWA and sorted, and PCR duplications were removed with GATK 4.0. Single nucleotide variants, insertions, and deletions were detected with Strelka2 with default parameters. Variants and polymorphisms were annotated with the Ensembl Variant Effect Predictor. Somatic copy number variations (CNVs) were analyzed with FACETS, and the resulting CNVs were used in further analyses.

### Statistical analysis

Chi-square tests or Fisher’s exact tests were used to compare the frequency data between 2 groups. Kappa consistency testing was applied to examine the consistency of MMR and MSI detection results. Receiver operator characteristic (ROC) curves were used to evaluate the performance of LS pre-screening methods. Statistical significance was defined by two-tailed *P* values < 0.05.

## Results

### The consistency of MSI testing and MMR IHC

**Figure 2:**
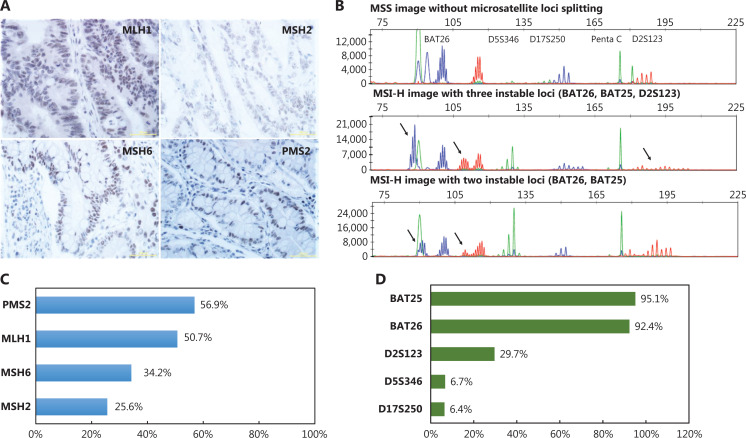
Results of MMR IHC and MSI testing. (A) Positive results of IHC staining for MLH1, MSH2, MSH6, and PMS2 (bar = 50 µm). (B) Representative figures of MSS and MSI-H results. (C) Incidence of loss of expression for each MMR protein in patients with dMMR. (D) Incidence of instability for each MSI locus in patients with MSI-H/L.

A total of 2,950 patients with CRC from the 5 medical centers were pre-screened with MMR IHC; 406 (13.8%) cases were determined to be dMMR tumors (**Figure 2A**). A total of 250 sex-matched cases among the patients with pMMR were used as a control cohort for further analysis (**Figure 1**). Among the 406 dMMR cases, 5 (1.2%) showed deficiency in 4 MMR proteins, 11 (2.7%) showed deficiency in 3 MMR proteins, 154 (37.9%) showed a loss of expression of MLH1 and PMS2 proteins, 72 (17.7%) showed a loss of expression of MSH6 and MSH2 proteins, and 153 (37.7%) showed deficiency in a single MMR protein. For the 4 MMR proteins, PMS2 loss occurred most frequently and was found in 56.9% of dMMR samples, followed by the loss of MLH1 (50.7%), MSH6 (34.2%), and MSH2 (25.6%) (**Figure 2C**).

We performed MSI testing on a total of 656 CRC samples, including 406 dMMR cases and 250 pMMR cases. Approximately 81.5% of dMMR cases were determined to be MSI-H/L (319 MSI-H and 12 MSI-L), and 75 (18.5%) were found to be microsatellite stable (MSS). Among the 250 pMMR cases, 246 (98.4%) were identified as MSS, whereas, only 2 (0.8%) were identified as MSI-H, and 2 (0.8%) were identified as MSI-L. Representative figures of MSS and MSI-H are displayed in **Figure 2B**. For the 5 microsatellite loci, BAT25 (95.1%) and BAT26 (92.4%) were most frequently positive, followed by D2S123 (29.7%), D17S250 (6.4%), and D5S346 (6.7%) in the MSI-H/L patients (**Figure 2D**). The consistency of MSI testing with MMR IHC in all enrolled patients with CRC was estimated, and the Kappa value was 0.758 (**Supplementary Table S1**).

### Identification of LS by detection of germline variants of MMR genes

Multi-gene panel NGS was performed on 656 cases, including 406 dMMR and 250 pMMR cases. Consequently, 154 cases were found to have at least one variant in MMR genes (*MLH1*, *MSH2*, *MSH6*, *PMS2*, or *EPCAM*) annotated as pathogenic or likely pathogenic, including 123 cases with 1 mutated MMR gene, 25 cases with 2 mutated MMR genes, and 6 cases with 3 mutated MMR genes (**Supplementary Table S2**).

**Figure 3:**
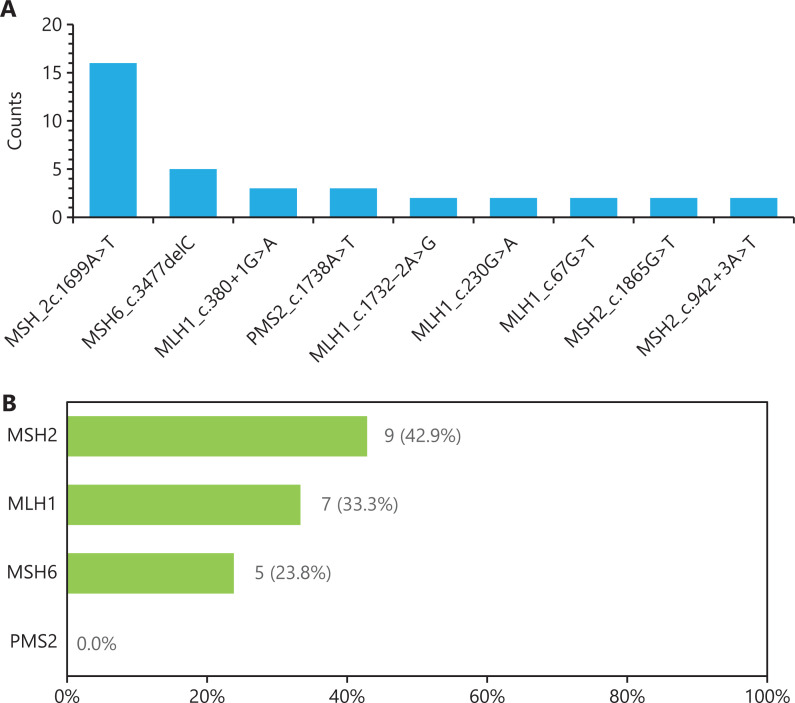
Distribution of germline alterations of MMR genes in LS. (A) Counts of repeated variants in Chinese patients with LS. (B) Incidence of 21 novel variants in each MMR gene.

Sanger sequencing was then performed on patients with MMR germline variants detected by NGS. Finally, 88 cases were confirmed as LS, and 38 cases were confirmed to be without germline variants according to Sanger sequencing; the other 28 cases were unclassified, owing to unidentified pathogenic or likely pathogenic variants. Among 88 LS cases, 85 cases had 1 mutated MMR gene, and 3 cases had 2 mutated MMR genes (**Supplementary Table S2**). Among the 96 pathogenic or likely pathogenic variants identified in 88 patients with LS, the variant frequency of *MLH1*, *MSH2*, *MSH6*, and *PMS2* was 37.5%, 44.3%, 14.8%, and 6.8%, respectively, in agreement with results reported in the literature^11^. A total of 46 types of alterations were annotated as pathogenic/likely pathogenic in the Clinvar Database. The c.1699A>T variant in MSH2 was the most frequent variant observed in Chinese patients with LS, with 16 repeated variants (**Figure 3A**). Meanwhile, 21 types of alterations were predicted as pathogenic and had not been collected in the Clinvar Database (**Table 1**).

**Table 1:** Novel germline variants of MMR genes in Chinese patients with CRC

Patient	Age (years)	Stage	Gene	Variant	Pathogenicity	Sanger sequencing	LS/sporadic CRCs	MMR IHC	MSI
P1	62	III	MLH1	c.1641dupA:p.Y548Ifs*9	Pathogenic	MLH1	LS	dMMR	MSI-H
P6	51	II	MLH1	c.304G>T:p.E102*	Pathogenic	MLH1	LS	dMMR	MSI-H
P8	41	III	MLH1	c.1852_1854delAAG:p.K618delK	Pathogenic	MLH1	LS	dMMR	MSI-H
P17	64	II	MLH1	c.1929_1938delTGACAACTAT:p.I643Mfs*15	Pathogenic	MLH1	LS	dMMR	MSI-H
P20	42	II	MLH1	c.1631_1634delAAAC:p.Q544Pfs*46	Pathogenic	MLH1	LS	dMMR	MSI-H
P23	49	II	MSH6	c.2104dupT:p.S702Ffs*4	Pathogenic	MSH6	LS	dMMR	MSI-H
P33	55	II	MSH6	c.1250delA:p.K417Sfs*36	Pathogenic	MSH6	LS	dMMR	MSI-H
P43	57	III	MSH6	c.1280delA:p.Y427Sfs*26	Pathogenic	MSH6	LS	dMMR	MSI-H
P48	65	II	MSH2	c.2251G>T:p.Gly751*	Pathogenic	MSH2	LS	dMMR	MSI-H
P52	64	II	MSH6	c.855_858delTGAG:p.S285Rfs*5	Pathogenic	MSH6	LS	dMMR	MSI-H
P56	26	II	MLH1	c.2107G>T:p.E703*	Pathogenic	MLH1	LS	dMMR	MSI-H
P72	56	II	MSH2	c.1978delG:p.D660Ifs*25	Pathogenic	MSH2	LS	dMMR	MSI-H
P82	56	II	MSH2	c.505delA:p.I169Yfs*5	Pathogenic	MSH2	LS	dMMR	MSI-H
P86	51	III	MLH1	c.753delC:p.S252Qfs*2	Pathogenic	MLH1	LS	dMMR	MSI-H
P91	52	II	MSH2	c.1981A>T:p.K661*	Pathogenic	MSH2	LS	dMMR	MSI-H
P119	46	I	MSH2	c.1472delA:p.K491Rfs*6	Pathogenic	MSH2	LS	dMMR	MSI-H
P124	48	II	MLH1	c.1852_1854delAAG:p.K618delK	Pathogenic	MLH1	LS	dMMR	MSI-H
P142	46	II	MSH6	c.3646G>T:p.Gly1216*	Pathogenic	MSH6	LS	dMMR	MSI-H
P143	45	I	MSH2	c.2588_2601del14:p.Y863Sfs*14	Pathogenic	MSH2	LS	dMMR	MSI-H
P152	59	II	MSH2	c.273dupT:p.L92Sfs*8	Pathogenic	MSH2	LS	dMMR	MSI-H
P152	59	II	MSH2	c.516delA:p.K172Nfs*2	Pathogenic	MSH2	LS	dMMR	MSI-H
P154	54	II	MSH2	c.643_644insG:p.Q215Rfs*17	Pathogenic	MSH2	LS	dMMR	MSI-H

MMR, mismatch repair; dMMR, deficient MMR; pMMR, proficient MMR; WT, wildtype; MSI, microsatellite instability; MSI-H, MSI-high; MSI-L, MSI-low.

These 21 variants were predicted to be pathogenic according to the American College of Medical Genetics and Genomics guidelines for variant interpretation. These variants were mainly stop-gain and frameshift variations. Most of the novel variants can lead to premature translation-termination codons, which trigger nonsense-mediated mRNA decay and elimination of MMR gene expression. Among them, 9 variants occurred in *MSH2* exons 2, 3, 9, 12, 14, and 15; 7 variants occurred in *MLH1* exons 3, 9, 14, 16, 17, and 19; 5 variants occurred in *MSH6* exons 4 and 7; and the MLH1 p.K618delK variant occurred in 2 patients (**Figure 3B**). Meanwhile, all 21 patients with these novel variants confirmed by Sanger sequencing presented dMMR and MSI-H in tumors. These results indicated that the 21 novel MMR germline variants can also be used to identify patients with LS. Finally, the patients were classified as having 88 LS and 540 sporadic CRC cases according to variants detected by NGS and Sanger sequencing. In addition, 28 patients were unclassified, owing to unidentified pathogenic or likely pathogenic variants determined by Sanger sequencing.

### Clinicopathologic features of LS in comparison with sporadic CRC

The comparisons of demographic and clinicopathologic features between LS (*n* = 88) and sporadic CRCs (*n* = 540) are shown in **Table 2**. The median age of patients with LS and sporadic CRCs was 53 (22–71) and 60 (23-86), respectively. However, most patients with LS (60.2%) were younger than 55 years of age, and most patients with sporadic CRCs (63.5%) were older than 55 years of age (*P* < 0.001). Compared with sporadic CRCs, LS had less advanced stages (*P* = 0.001) and occurred more frequently on the right side (67.5% *vs.* 39.4%, *P* < 0.001), i.e., from the cecum to the splenic flexure. Mucinous adenocarcinoma was much more common in LS (66.7%) than in sporadic CRCs (29.3%) (*P* < 0.001). Demographic and clinicopathologic features were also compared among LS (*n* = 88), dMMR/MSI-H sporadic CRCs (*n* = 292), and non-dMMR/MSI-H sporadic CRCs (*n* = 248) (**Supplementary Table S3**). Patients with LS had significantly different ages from those with dMMR/MSI-H sporadic CRCs, but differences were not observed in the clinical stage, location, degree of differentiation, or mucinous histological subtype. However, non-dMMR/MSI-H sporadic CRCs significantly differed from dMMR/MSI-H sporadic CRCs in clinical stage, location, degree of differentiation, and mucinous histological subtype. In addition, demographic and clinicopathologic feature comparisons among LS subgroups with different germline variant genes, and LS subgroups with sporadic CRCs, are shown in **Supplementary Tables S4** and **S5**. Patients with LS with the MLH1 variant, MSH2 variant, and MSH6 variant showed similar trends to all patients with LS in terms of age, clinical stage, location, mucinous histological subtype, MSI, and dMMR, although some subgroups showed no significant differences, owing to the limited number of patients. Patients with LS with PMS2 variants also showed similar trends to those of all patients with LS in these clinical factors, except for age. No significant differences were observed among LS subgroups with different germline mutated genes, except for the PMS2 variant LS subgroup. The observed features of the PMS2 variant group might have been due to the very limited number of patients in this subgroup.

**Table 2:** Demographic and clinicopathological features of LS and sporadic CRCs

Parameters	Total, *n* (%) *n* = 628	LS, *n* (%) *n* = 88	Sporadic, *n* (%) *n* = 540	*P* value
Gender				0.48
Male	362 (57.9%)	54 (61.4%)	308 (57.4%)
Female	263 (42.1%)	34 (38.6%)	229 (42.6%)
Age (years)				<0.001
Median (range)	59 (22–86)	53 (22–71)	60 (23–86)
<55	250 (39.8%)	53 (60.2%)	197 (36.5%)
≥55	378 (60.2%)	35 (39.8%)	343 (63.5%)
Clinical stage				0.001
I	55 (9.6%)	11 (13.6%)	44 (8.9%)
II	282 (49.2%)	53 (65.4%)	229 (46.5%)
III	203 (35.4%)	15 (18.5%)	188 (38.2%)
IV	33 (5.8%)	2 (2.5%)	31 (6.3%)
Location				<0.001
Left	321 (56.6%)	26 (32.5%)	295 (60.6%)
Right	246 (43.4%)	54 (67.5%)	192 (39.4%)
Differentiation				0.051
Low	114 (22.6%)	21 (33.3%)	93 (21.0%)
Medium	369 (73.1%)	38 (60.3%)	331 (74.9%)
High	22 (4.4%)	4 (6.3%)	18 (4.1%)
Mucinous				<0.001
No	292 (68.1%)	10 (33.3%)	282 (70.7%)
Yes	137 (31.9%)	20 (66.7%)	117 (29.3%)
MSI				<0.001
MSI-H	301 (47.9%)	80 (90.9%)	221 (40.9%)
MSI-L	12 (1.9%)	1 (1.1%)	11 (2.0%)
MSS	315 (50.2%)	7 (8.0%)	308 (57.0%)
MMR-IHC				<0.001
dMMR	378 (60.2%)	87 (98.9%)	291 (53.9%)
pMMR	250 (39.8%)	1 (1.1%)	249 (46.1%)

MMR, mismatch repair; dMMR, deficient MMR; pMMR, proficient MMR; MSI, microsatellite instability; MSI-H, MSI-high; MSI-L, MSI-low; MSS, microsatellite stability.

### Comparison of MSI testing and MMR IHC in LS pre-screening

**Figure 4:**
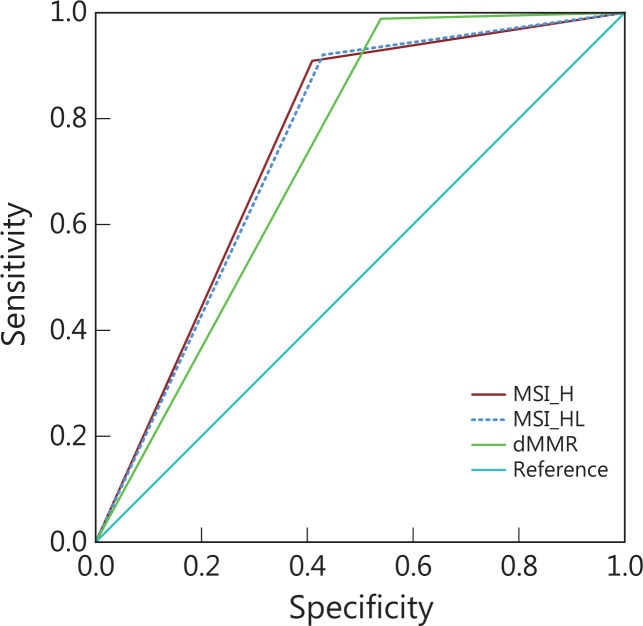
Performance of LS pre-screening methods, evaluated with ROC curves of MSI-H, MSI-H/L, and dMMR. AUC, area under curve.

Pre-screening of LS probands in patients with CRC by MMR IHC and/or MSI testing is strongly recommended in the NCCN Clinical Practice Guidelines. The performance of MMR IHC, and MSI testing for LS pre-screening was compared in our cohort, and the detailed efficiency is displayed in **Supplementary Table S6** and **Figure 4**. Briefly, the specificity and positive predictive value (PPV) of MSI testing (59.1%/57.0% and 26.6%/25.9%) were slightly higher than those of MMR IHC (46.1% and 23.0%), whereas the sensitivity of MSI testing (90.0%/92.0%) was slightly lower than that of MMR IHC (98.9%). Overall, the performance of MMR IHC and MSI testing for LS pre-screening was comparable, with high sensitivity and high negative predictive value (NPV), but low PPV (<30%). The ROC curves for dMMR, MSI-H, and MSI-H/L are shown in **Figure 4**. The area under the ROC curve (AUC) for dMMR, MSI-H, and MSI-H/L was 0.725, 0.750, and 0.745, respectively.

### Genetic differences between patients with dMMR_MSI-H LS and dMMR_MSI-H sporadic CRCs

To enhance the PPV of MMR and MSI testing for LS pre-screening, we attempted to determine the genetic differences between patients with LS with dMMR_MSI-H and sporadic CRCs with dMMR_MSI-H. We performed WES on 14 cases of LS with dMMR_MSI-H and 15 sporadic CRCs with dMMR_MSI-H.

**Figure 5:**
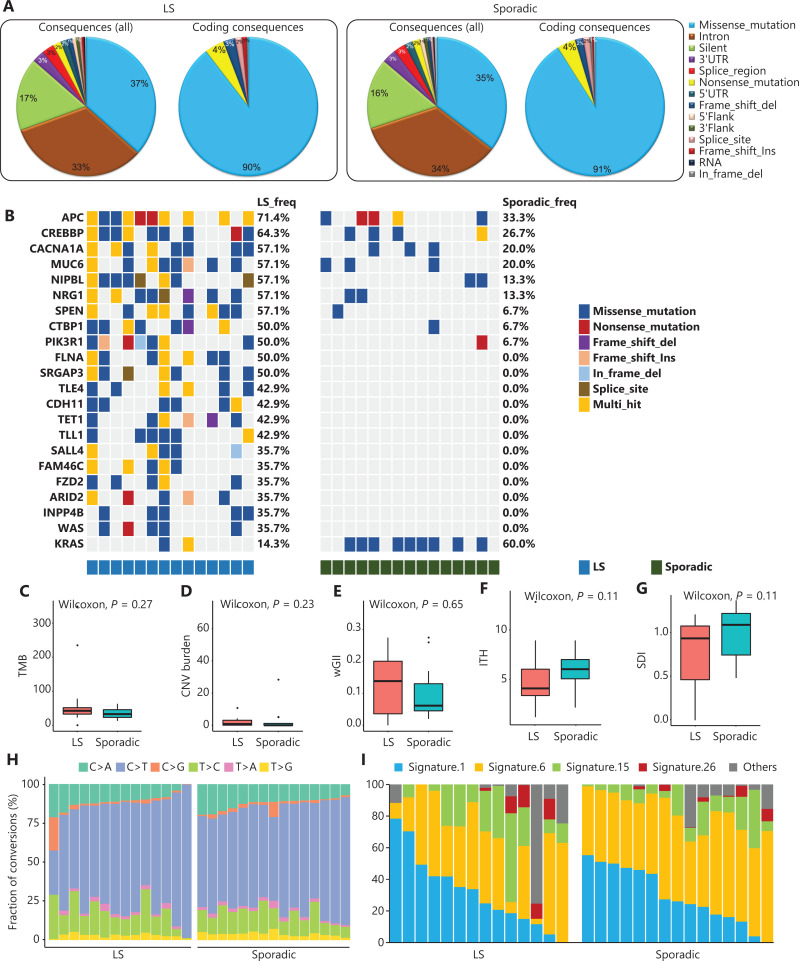
Comparison of genetic features between patients with dMMR_MSI-H LS and dMMR_MSI-H sporadic CRCs. (A) Variant analysis based on whole genetic variants identified by the Strelka2 pipeline. (B) Cancer associated genes with significantly different variant frequencies between patients with LS and sporadic CRCs. Each row represents a gene, each column represents a sample, and colors represent types of variants. TMB (C), CNV burden (D), wGII (E), ITH index (F), SDI (G), base substitutions (H), and mutational signatures (I) in patients with LS and sporadic CRCs.

A total of 15,066 mutated genes were detected in all samples. LS and sporadic CRCs had similar variant distributions, thus showing that missense mutation and intron were predominant (**Figure 5A**). Among them, some cancer associated genes showed significantly different variant frequencies between LS and sporadic CRCs, including driver genes and genes in cancer related pathways (**Figure 5B**). As shown, most of these genes had significantly higher variant frequency in LS than sporadic CRC. Notably, the variant frequencies of *APC* and *CREBBP* exceeded 60%, whereas variants of *FLNA*, *SRGAP3*, *TLE4*, *CDH11*, *TET1*, *TLL1*, *SALL4*, *FAM46C*, *FZD2*, *ARID2*, *INPP4B*, and *WAS* were found exclusively in LS CRCs. Interestingly, the variant frequency of *KARS* was significantly higher in sporadic CRCs (reaching 60%) than LS. The median TMB of patients with LS (44.4 mutations/Mb) was higher than that of patients with sporadic CRCs (33.2 mutations/Mb), but the difference was not significant (**Figure 5C**). Thus, these LS and sporadic CRC associated genes, including highly mutated *APC*, *CREBBP*, and *KRAS*, may have the potential to distinguish dMMR_MSI-H LS from sporadic CRCs.

Different aspects of genetic characteristics were also compared between groups. The median CNV burden and wGII of patients with LS (0.29 and 0.14, respectively) were higher than those of patients with sporadic CRCs (0.01 and 0.06, respectively) (**Figure 5D** and **5E**). However, the CNV burdens of both groups were much lower than those for other types of tumors, including renal cancer and prostate cancer^18,19^. Compared with patients with sporadic CRCs, patients with LS had slightly lower intratumoral heterogeneity (ITH), as illustrated by the median ITH index (4 *vs.* 6) and the median Shannon diversity index (SDI) (1.0 *vs.* 1.1) (**Figure 5F** and **5G**). These differences in CNV burden, wGII, ITH index, SDI, base substitutions, or mutational signatures were not significant between LS and sporadic CRCs. In both groups, C>T transitions were dominant, followed by T>C transitions and C>A transversions (**Figure 5H**). Mutational signature 1, which is associated with age at cancer diagnosis, and signature 6, which is associated with defective DNA mismatch repair, were prevalent in both patients with LS and patients with sporadic CRCs. Moreover, signature 15, which is associated with defective DNA mismatch repair and high numbers of small insertions and deletions at mono/polynucleotide repeats, was more often observed in patients with LS than sporadic CRCs, although no significant difference was observed (**Figure 5I**).

Different aspects of genetic characteristics were also compared among groups with other different clinical characteristics. Several cancer related genes with different variant frequencies in groups with different clinical characteristics were observed. As shown in **Supplementary Figure S1**, the variant frequencies of *FN1*, *BAP1*, *NOTCH2*, *ARHGEF12*, *ARID1B*, *USP6*, and *ZNF780A* were significantly lower in patients with CRC who were <55 years of age. The variant frequency of *CREBBP* was significantly higher in patients with early-stage CRC, whereas those of *AFF3* and *BRAF* were significantly lower. The variant frequencies of *NRG1*, *FLNA*, *PTCH1*, *SPEN*, and *TET1* were significantly higher in patients with left CRC. The variant frequencies of *CTNND1*, *FOXA2*, and *CNOT3* were significantly higher in patients with low differentiated CRC. In addition, the TMB, CNV burden, wGII, ITH index, and SDI showed no significant differences according to age, stage, location, or degree of differentiation. No significantly different mutational signatures were observed in groups with different ages, stages, or locations, whereas signatures 6 and 15 were more prevalent in moderately and highly differentiated groups (**Supplementary Figure S2**). These results indicated several somatic variant genes and mutational signatures associated with clinical features.

**Figure 6:**
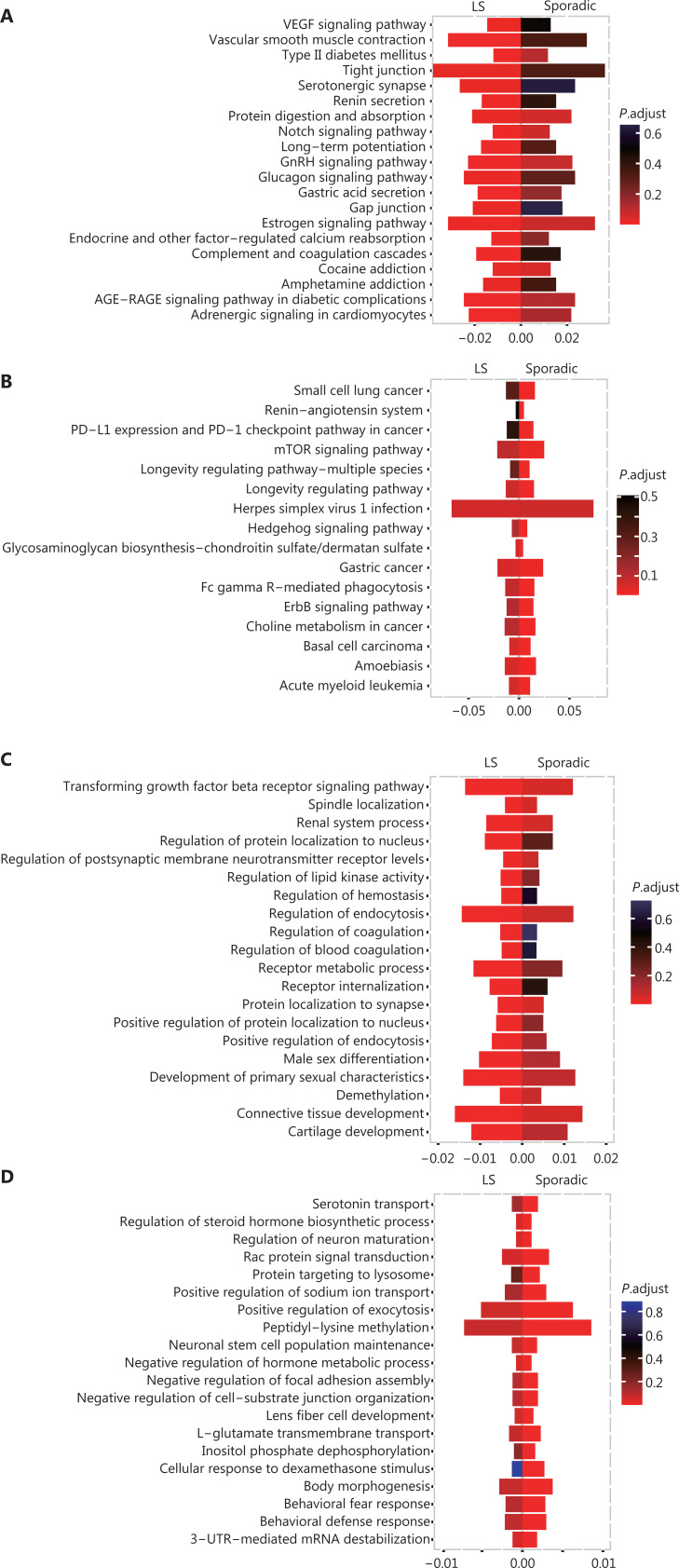
Differences in enriched pathways between patients with LS and sporadic CRC. (A) Pathways significantly enriched only in LS rather than in sporadic CRCs, according to KEGG analysis. (B) Pathways significantly enriched only in sporadic CRCs rather than in LS, according to KEGG analysis. (C) Pathways significantly enriched only in LS rather than in sporadic CRCs, according to GO biological process analysis. (D) Pathways significantly enriched only in sporadic CRCs rather than LS, according to GO biological process analysis.

We further explored the differences between patients with LS and sporadic CRCs by using GO and KEGG clustering analysis. Although several common biological processes and pathways were found, many unique enriched pathways were also observed. The LS-unique significantly enriched biological processes and pathways are shown in **Figure 6A** and **6C**, including the VEGF signaling pathway, Notch signaling pathway, and transforming growth factor beta receptor (TGFβR) signaling pathway. The sporadic CRC-unique significantly enriched biological processes and pathways are shown in **Figure 6B** and **6D**, including the mTOR signaling pathway, ErbB signaling pathway, and Rac protein signal transduction.

## Discussion

In this large multi-center cohort study, we evaluated the consistency of MMR IHC and MSI testing, and compared the performance of MMR IHC and MSI testing in LS pre-screening. Among 2950 total CRCs, 406 (13.8%) were dMMR. Among 406 dMMR and 250 matched pMMR controls, 321 were MSI-H, and 14 were MSI-L. Among them, 154 cases had pathogenic or likely pathogenic germline variants in MMR genes detected by NGS, and 88 (57.1%) cases were confirmed by Sanger sequencing, thus indicating that NGS is effective in LS screening. The variant frequencies of *MLH1*, *MSH2*, *MSH6*, and *PMS2* were 37.5%, 44.3%, 14.8%, and 6.8%, respectively, in agreement with previous reports^20,21^. To our knowledge, this is the first large multi-center cohort study performing LS identification in Chinese patients with CRC through multi-gene panel NGS, and also including MMR IHC and MSI testing for pre-screening, and comparison of systematic somatic genetic characteristics between LS and sporadic CRCs with dMMR_MSI-H. A single-center study of patients with CRC from Southeast China has identified 2.8% of CRCs as LS by MMR IHC plus *BRAF* screening and sequential germline sequencing^22^.

The performance of MMR IHC and MSI testing in LS pre-screening was comparable, with high sensitivity and high NPV but low PPV (<30%). Moreover, ROC analysis showed that the AUC values for MSI-H and MSI-H/L were slightly higher than that for dMMR, i.e., 0.750 and 0.745 *vs.* 0.725. These results indicated that MSI testing may have a technical advantage over MMR IHC; this finding warrants further attention and exploration in LS pre-screening. Although the PPV values for MSI-H and MSI-H/L were slightly higher than that for dMMR, i.e., 26.6% and 25.9% *vs.* 23.0%, the PPV values were all low. To enhance the performance of MSI and dMMR testing for LS pre-screening, combination with other clinical and somatic genetic characteristics is necessary to improve the performance of LS pre-screening.

MSI, dMMR, age, clinical stage, tumor location, and mucinous type showed significant differences between LS and sporadic CRCs, thus indicating that these factors had significant associations with LS. These relationships are supported by previous reports^23–25^. Family history has also been reported to be associated with LS CRCs in previous studies^17,23^. The combination of these LS related clinical features with MSI and dMMR may further improve the performance of LS pre-screening.

Using WES analysis, we observed substantial differences in somatic genetic characteristics between patients with dMMR_MSI-H LS and dMMR_MSI-H sporadic CRCs. Highly mutated *APC* and *KRAS* showed significantly different mutational frequencies between patients with dMMR_MSI-H LS and dMMR_MSI-H sporadic CRCs. They have also been reported to be highly mutated and to play key roles in CRCs^26,27^. The LS unique mutated genes included *TLL1*, *SALL4*, *FAM46C*, *FZD2*, *ARID2*, *INPP4B*, and *WAS*. Among them, *SALL4* has been reported to be associated with the progression and metastasis of CRCs^28^, and *ARID2* is frequently mutated in microsatellite unstable CRC^29^. Although genetic characteristics of CRCs have been widely reported^30–33^, studies performing systematic comparison of somatic genetic characteristics between LS and sporadic CRCs with dMMR_MSI-H have been very limited. Some research has shown that *APC* mutations commonly occur after the onset of MMR deficiency, in support of the different variant frequencies of *APC* in this study^34^. A study on the relationship between the APC mutation frequency and LS has received substantial attention^35,36^. To our knowledge, the present work is the first investigation revealing that VEGF and Notch pathways are uniquely enriched in LS CRCs but not enriched in sporadic CRCs with dMMR_MSI-H. However, the mTOR signaling pathway and ErbB pathways were uniquely enriched in sporadic CRCs. The ErbB pathway difference was supported by a study indicating that the addition of anti-EGFR to chemotherapy is associated with different effects in progression-free survival in familial or sporadic MSI CRCs^37^. The higher median TMB of patients with LS in this study was consistent with findings from previous studies^18,19^. Insignificant differences might have been due to the limited samples analyzed by WES. These differences may contribute to distinguishing LS from sporadic CRCs, and the combination of these LS related genetic features with MSI and dMMR may further improve the performance of LS pre-screening.

Our study has several limitations. First, the efficacy, particularly the specificity of MMR IHC and MSI testing for LS pre-screening of tumor samples, may be improved by excluding patients with dMMR/MSI-H caused by methylation of the *MLH1* promoter or double somatic MMR variants^13,38^. We assessed the efficacy of only dMMR and MSI-H as pre-screening approaches, because MLH1 promoter methylation was not tested, and the samples with somatic variants were limited in this study. Second, some LS cases might have been missed by the germline MMR gene panel NGS, which cannot detect large deletions in MMR genes. For patients with dMMR/MSI-H but without germline MMR variants detected by targeted NGS, if strong hereditary clinical criteria are met, further genetic screening is recommended. Clinicians importantly must interpret the results of germline MMR gene panel NGS in the context of family history. Third, family history and personal tumor disease history information were not collected in this study, but might have further supported the definition of LS and improved the LS pre-screening. Additional studies with more patients and more comprehensive information are needed to validate and improve on these results.

## Conclusions

In conclusion, MMR IHC and MSI testing are comparable and effective methods for LS pre-screening. A total of 21 novel pathologic variants in MMR genes were found in Chinese patients with CRCs. Compared with patients with sporadic CRCs, patients with LS were younger. Tumors of patients with LS frequently occurred on the right side, were found in early stages, and were mucinous. Substantial differences in somatic genetic characteristics were observed between patients with dMMR_MSI-H LS and dMMR_MSI-H sporadic CRCs. Clinical and somatic genetic characteristics may have the potential to distinguish dMMR_MSI-H LS from dMMR_MSI-H sporadic CRCs. In addition, the somatic genetic characteristics of dMMR_MSI-H LS and sporadic CRCs provided important information for potential therapy for patients with CRC.
