# Supplementary material for: Lynch syndrome pre-screening and comprehensive characterization in a multi-center large cohort of Chinese patients with colorectal cancer
Source: Cancer Biol Med. 2022 Jun 1;19(8):1235–48. doi: 10.20892/j.issn.2095-3941.2021.0585 (PMC9425183; doi:10.20892/j.issn.2095-3941.2021.0585)
Supplement: Supplementary file 1 [file cbm-19-1235-s001.pdf]

Supplementary materials

Table S1 Consistency of MMR-IHC and MSI testing

	MSS	MSI-H/L	Total	Kappa
pMMR	246 (98.4%)	4 (1.6%)	250	0.758
dMMR	75 (18.5%)	331 (81.5%)	452	
Total	321	335	656	

MMR, mismatch repair; dMMR, deficient MMR; pMMR, proficient MMR; MSI, microsatellite instability; MSI-H, MSI-high; MSI-L, MSI-low; MSS, microsatellite stability.

Table S2 Germline variants of MMR genes detected by NGS and Sanger sequencing

Patient	Age (years)	Stage	Gene	Variant	Pathogenicity	Sanger sequencing	LS/sporadic CRCs	MMR_IHC	MSI
P1	62	III	MLH1	c.1641dupA:p.Y548Ifs*9	Pathogenic	MLH1	LS	dMMR	MSI-H
P2	58	II	MSH2	c.2548G>T:p.E850*	Pathogenic	WT	Sporadic	dMMR	MSI-H
P3	46	III	MLH1	c.826dupA:p.I276Nfs*31	Pathogenic	Unidentified	Unclassified	dMMR	MSI-H
P4	54	III	MSH2	c.1386+1G>A	Likely pathogenic	MSH2	LS	dMMR	MSI-H
P5	46	II	MLH1	c.230G>A:p.Cys77Tyr	Pathogenic	MLH1	LS	dMMR	MSI-H
P5	46	II	MLH1	c.585_588+4delAAAAGTAA	Pathogenic	Unidentified	LS	dMMR	MSI-H
P6	51	II	MLH1	c.304G>T:p.E102*	Pathogenic	MLH1	LS	dMMR	MSI-H
P7	61	II	MLH1	c.1896+1G>A:c.1896+2T>C	Likely pathogenic	MLH1	LS	dMMR	MSI-H
P8	41	III	MLH1	c.1852_1854delAAG:p.K618delK	Pathogenic	MLH1	LS	dMMR	MSI-H
P8	41	III	MLH1	c.237_238insACTGCAG:p.F80Tfs*6	Pathogenic	Unidentified	LS	dMMR	MSI-H
P9	60	II	PMS2	c.2174+1G>A	Pathogenic	PMS2	LS	dMMR	MSI-H
P10	61	NA	PMS2	c.1970delA:p.Asn657Ilefs	Likely pathogenic	WT	Sporadic	dMMR	MSI-H
P11	61	III	MSH6	c.2845dupC:p.Q949Pfs*17	Pathogenic	Unidentified	Unclassified	dMMR	MSI-H
P12	60	II	PMS2	c.1738A>T:p.Lys580Ter	Pathogenic	PMS2	LS	dMMR	MSI-H
P13	45	II	MLH1	c.791-9_792dupATTGTTTAGAT	Likely pathogenic	Unidentified	Unclassified	dMMR	MSI-H
P14	64	III	MSH2	c.166delG:p.E56Rfs*8	Pathogenic	MSH2	LS	dMMR	MSI-H
P15	49	I	MLH1	c.67G>T:p.Glu23Ter	Pathogenic	MLH1	LS	dMMR	MSI-H
P16	44	II	MSH2	c.2131C>T:p.Arg711Ter	Pathogenic	MSH2	LS	dMMR	MSI-H
P17	64	II	MLH1	c.1929_1938delTGACAACTAT:p.I643Mfs*15	Pathogenic	MLH1	LS	dMMR	MSI-H
P18	61	I	PMS2	c.1970delA:p.Asn657Ilefs	Likely pathogenic	WT	Sporadic	pMMR	MSS
P19	25	NA	MLH1	c.67G>T:p.Glu23Ter	Pathogenic	MLH1	LS	dMMR	MSI-H
P19	25	NA	PMS2	c.1970delA:p.Asn657Ilefs	Likely pathogenic	WT	LS	dMMR	MSI-H
P19	25	NA	MLH1	c.229_230insC:p.C77Sfs*2	Pathogenic	Unidentified	LS	dMMR	MSI-H
P20	42	II	MLH1	c.1631_1634delAAAC:p.Q544Pfs*46	Pathogenic	MLH1	LS	dMMR	MSI-H

Table S2 Continued

Patient	Age (years)	Stage	Gene	Variant	Pathogenicity	Sanger sequencing	LS/sporadic CRCs	MMR_IHC	MSI
P21	45	II	MSH2	c.1699A>T:p.Lys567Ter	Pathogenic	MSH2	LS	dMMR	MSI-H
P22	46	I	MSH6	c.3013C>T:p.Arg1005Ter	Pathogenic	MSH6	LS	dMMR	MSS
P22	46	I	MLH1	c.677+2T>C	Likely pathogenic	MLH1	LS	dMMR	MSS
P23	49	II	MSH6	c.2104dupT:p.S702Ffs*4	Pathogenic	MSH6	LS	dMMR	MSI-H
P24	52	III	PMS2	c.1970delA:p.Asn657Ilefs	Likely pathogenic	WT	Sporadic	dMMR	MSS
P25	58	II	MLH1	c.677G>A:p.Arg226Gln	Pathogenic	MLH1	LS	dMMR	MSI-H
P26	62	II	PMS2	c.564dupA:p.H189Tfs*60	Pathogenic	WT	Sporadic	dMMR	MSS
P27	51	II	MLH1	c.230G>A:p.Cys77Tyr	Pathogenic	MLH1	LS	dMMR	MSI-H
P28	46	II	MLH1	c.2080G>T:p.E694*	Likely pathogenic	MLH1	LS	dMMR	MSI-L
P29	56	NA	PMS2	c.1970delA:p.Asn657Ilefs	Likely pathogenic	WT	Sporadic	dMMR	MSI-H
P30	60	II	MSH2	c.1699A>T:p.Lys567Ter	Pathogenic	MSH2	LS	dMMR	MSI-H
P31	62	II	MSH2	c.1699A>T:p.Lys567Ter	Pathogenic	Unidentified	Unclassified	dMMR	MSI-H
P32	73	III	PMS2	c.1970delA:p.Asn657Ilefs	Likely pathogenic	WT	Sporadic	dMMR	MSS
P33	55	II	MSH6	c.1250delA:p.K417Sfs*36	Pathogenic	MSH6	LS	dMMR	MSI-H
P34	69	I	MSH2	c.942+3A>T	Pathogenic	MSH2	LS	dMMR	MSS
P35	56	II	MSH2	c.1427delC:p.P476Lfs*6	Pathogenic	Unidentified	Unclassified	dMMR	MSI-L
P36	50	III	MSH6	c.718C>T:p.Arg24Ter	Pathogenic	WT	Sporadic	dMMR	MSI-H
P36	50	III	PMS2	c.1970delA:p.Asn657Ilefs	Likely pathogenic	WT	Sporadic	dMMR	MSI-H
P37	85	IV	MSH2	c.942+3A>T	Pathogenic	Unidentified	Unclassified	dMMR	MSS
P37	85	IV	MSH6	c.2104dupT:p.S702Ffs*4	Pathogenic	Unidentified	Unclassified	dMMR	MSS
P37	85	IV	PMS2	c.1136dupA:p.D379Efs*3	Pathogenic	Unidentified	Unclassified	dMMR	MSS
P38	57	II	MSH2	c.1699A>T:p.Lys567Ter	Pathogenic	MSH2	LS	dMMR	MSI-H
P39	76	NA	PMS2	c.1970delA:p.Asn657Ilefs	Likely pathogenic	WT	Sporadic	dMMR	MSI-H
P40	75	II	PMS2	c.1970delA:p.Asn657Ilefs	Likely pathogenic	WT	Sporadic	dMMR	MSS
P41	77	I	PMS2	c.1970delA:p.Asn657Ilefs	Likely pathogenic	WT	Sporadic	dMMR	MSS
P42	63	NA	PMS2	c.1970delA:p.Asn657Ilefs	Likely pathogenic	WT	Sporadic	dMMR	MSI-H
P43	57	III	MSH6	c.1280delA:p.Y427Sfs*26	Pathogenic	MSH6	LS	dMMR	MSI-H
P44	62	III	PMS2	c.1970delA:p.Asn657Ilefs	Likely pathogenic	WT	Sporadic	dMMR	MSS
P45	56	II	MSH6	c.3477delC:p.Tyr1159Terfs	Pathogenic	MSH6	LS	dMMR	MSI-H
P45	56	II	MSH2	c.2228C>G:p.Ser743Ter	Pathogenic	MSH2	LS	dMMR	MSI-H
P46	44	III	MSH2	c.1699A>T:p.Lys567Ter	Pathogenic	MSH2	LS	dMMR	MSI-H
P47	44	II	MLH1	c.1668-2A>C	Likely pathogenic	MLH1	LS	dMMR	MSI-H
P48	65	II	MSH2	c.2251G>T: p.Gly751*	Pathogenic	MSH2	LS	dMMR	MSI-H
P49	80	II	PMS2	c.1970delA:p.Asn657Ilefs	Likely pathogenic	WT	Sporadic	dMMR	MSS
P50	54	III	MLH1	c.117-1G>A	Pathogenic	Unidentified	Unclassified	dMMR	MSI-H

Table S2 Continued

Patient	Age (years)	Stage	Gene	Variant	Pathogenicity	Sanger sequencing	LS/sporadic CRCs	MMR_IHC	MSI
P51	51	II	MSH2	c.1216C>T:p.Arg406Ter	Pathogenic	MSH2	LS	dMMR	MSI-H
P52	64	II	MSH6	c.855_858delTGAG:p.S285Rfs*5	Pathogenic	MSH6	LS	dMMR	MSI-H
P53	45	III	MLH1	c.678-2A>G	Likely pathogenic	Unidentified	Unclassified	dMMR	MSS
P54	59	NA	MLH1	c.67G>T:p.Glu23Ter	Pathogenic	WT	Sporadic	dMMR	MSS
P55	55	II	MSH6	c.1243C>T:p.Gln415Ter	Pathogenic	WT	Sporadic	dMMR	MSI-L
P56	26	II	MLH1	c.2107G>T:p.E703*	Pathogenic	MLH1	LS	dMMR	MSI-H
P57	35	II	MLH1	c.884+4A>G	Pathogenic	MLH1	LS	dMMR	MSI-H
P58	28	NA	MSH2	c.2211-2A>C	Likely pathogenic	MSH2	LS	dMMR	MSI-H
P58	28	NA	PMS2	c.1970delA:p.Asn657Ilefs	Likely pathogenic	WT	LS	dMMR	MSI-H
P59	39	II	MSH2	c.1865C>T:p.Pro622Leu	Pathogenic	MSH2	LS	dMMR	MSI-H
P60	34	II	MSH2	c.1147C>T:p.Arg383Ter	Pathogenic	MSH2	LS	dMMR	MSI-H
P61	53	II	MLH1	c.793C>A:p.Arg265Ser	Pathogenic	MLH1	LS	dMMR	MSI-H
P62	42	II	MSH6	c.742C>T:p.Arg248Ter	Pathogenic	Unidentified	Unclassified	dMMR	MSS
P63	54	II	MLH1	c.2041G>A:p.Ala681Thr	Pathogenic	MLH1	LS	dMMR	MSI-H
P64	56	NA	PMS2	c.1970delA:p.Asn657Ilefs	Likely pathogenic	WT	Sporadic	dMMR	MSI-H
P65	58	III	MSH6	c.2194C>T:p.Arg732Ter	Pathogenic	MSH6	LS	dMMR	MSI-H
P66	67	NA	MSH2	c.1203dupA:p.Q402Tfs*15	Pathogenic	WT	Sporadic	dMMR	MSS
P66	67	NA	PMS2	c.1970delA:p.Asn657Ilefs:c.1165C> T:p.Arg389Ter	Likely pathogenic	WT	Sporadic	dMMR	MSS
P67	31	III	MSH2	c.1699A>T:p.Lys567Ter	Pathogenic	MSH2	LS	dMMR	MSI-H
P68	60	I	MSH2	c.212-2A>G	Likely pathogenic	Unidentified	Unclassified	dMMR	MSI-H
P69	56	II	MSH2	c.862C>T:p.Gln288Ter	Pathogenic	MSH2	LS	dMMR	MSI-H
P70	54	NA	MSH2	c.1165C>T:p.Arg389Ter	Pathogenic	WT	Sporadic	dMMR	MSI-H
P70	54	NA	PMS2	c.1970delA:p.Asn657Ilefs	Likely pathogenic	WT	Sporadic	dMMR	MSI-H
P71	62	I	PMS2	c.1970delA:p.Asn657Ilefs	Likely pathogenic	WT	Sporadic	dMMR	MSS
P72	56	II	MSH2	c.1978delG:p.D660Ifs*25	Pathogenic	MSH2	LS	dMMR	MSI-H
P73	31	II	MSH2	c.1699A>T:p.Lys567Ter	Pathogenic	MSH2	LS	dMMR	MSI-H
P74	43	II	MSH2	c.1865C>T:p.Pro622Leu; c.1699A> T:p.Lys567Ter	Pathogenic	MSH2	LS	dMMR	MSI-H
P75	69	II	PMS2	c.1970delA:p.Asn657Ilefs	Likely pathogenic	WT	Sporadic	dMMR	MSS
P76	47	I	MLH1	c.791-1G>A	Pathogenic	MLH1	LS	dMMR	MSS
P77	67	NA	PMS2	c.1970delA:p.Asn657Ilefs	Likely pathogenic	WT	Unclassified	dMMR	MSI-H
P77	67	NA	PMS2	c.444delC:p.Y149Tfs*52	Pathogenic	Unidentified	Unclassified	dMMR	MSI-H
P78	70	II	MLH1	c.1975C>T:p.Arg659Ter	Pathogenic	WT	Sporadic	dMMR	MSS
P78	70	II	PMS2	c.1970delA:p.Asn657Ilefs	Likely pathogenic	WT	Sporadic	dMMR	MSS
P78	70	II	MLH1	c.476_477delTA:p.I159Sfs*12	Pathogenic	WT	Sporadic	dMMR	MSS

Table S2 Continued

Patient	Age (years)	Stage	Gene	Variant	Pathogenicity	Sanger sequencing	LS/sporadic CRCs	MMR_IHC	MSI
P79	74	II	MSH2	c.1699A>T:p.Lys567Ter	Pathogenic	Unidentified	Unclassified	dMMR	MSI-H
P80	36	I	MLH1	c.1683C>G:p.Tyr561Ter	Pathogenic	MLH1	LS	dMMR	MSI-H
P81	63	III	MSH2	c.1777C>T:p.Gln593Ter	Pathogenic	Unidentified	Unclassified	dMMR	MSS
P81	63	III	PMS2	c.1970delA:p.Asn657Ilefs	Likely pathogenic	WT	Unclassified	dMMR	MSS
P82	56	II	MSH2	c.505delA:p.I169Yfs*5	Pathogenic	MSH2	LS	dMMR	MSI-H
P83	53	III	MSH6	c.1610_1613delAGTA:p.K537Ifs*33	Pathogenic	WT	Sporadic	dMMR	MSS
P84	63	II	MSH2	c.1699A>T:p.Lys567Ter	Pathogenic	MSH2	LS	dMMR	MSI-H
P85	49	II	MLH1	c.1732-2A>G	Likely pathogenic	MLH1	LS	dMMR	MSS
P85	49	II	PMS2	c.1738A>T:p.Lys580Ter	Pathogenic	WT	LS	dMMR	MSS
P86	51	III	MLH1	c.753delC:p.S252Qfs*2	Pathogenic	MLH1	LS	dMMR	MSI-H
P87	55	II	PMS2	c.1738A>T:p.Lys580Ter	Pathogenic	PMS2	LS	dMMR	MSI-H
P88	73	NA	PMS2	c.1970delA:p.Asn657Ilefs	Likely pathogenic	WT	Sporadic	dMMR	MSI-H
P89	69	I	PMS2	c.1738A>T:p.Lys580Ter	Pathogenic	PMS2	LS	dMMR	MSI-H
P90	56	I	PMS2	c.232_235delGAAA:p.E78Tfs*5	Pathogenic	PMS2	LS	dMMR	MSI-H
P91	52	II	MSH2	c.1699A>T:p.Lys567Ter	Pathogenic	MSH2	LS	dMMR	MSI-H
P91	52	II	MSH2	c.1981A>T:p.K661*	Pathogenic	MSH2	LS	dMMR	MSI-H
P92	65	I	MSH2	c.518T>G:p.Leu173Arg	Pathogenic	Unidentified	Unclassified	dMMR	MSI-H
P93	54	II	MSH6	c.3477delC:p.Tyr1159Terfs	Pathogenic	MSH6	LS	dMMR	MSI-H
P94	53	II	MLH1	c.1783_1784delAG:p.S595Wfs*14	Pathogenic	MLH1	LS	dMMR	MSI-H
P95	58	II	MSH2	c.1165C>T:p.Arg389Ter	Pathogenic	MSH2	LS	dMMR	MSI-H
P96	62	II	MSH2	c.1699A>T:p.Lys567Ter	Pathogenic	MSH2	LS	dMMR	MSI-H
P97	46	II	MSH2	c.1699A>T:p.Lys567Ter	Pathogenic	MSH2	LS	dMMR	MSI-H
P97	46	II	MLH1	c.677+2T>C	Likely pathogenic	Unidentified	LS	dMMR	MSI-H
P98	54	II	MSH6	c.3477delC:p.Tyr1159Terfs	Pathogenic	MSH6	LS	dMMR	MSS
P99	46	II	MSH2	c.1699A>T:p.Lys567Ter	Pathogenic	Unidentified	Unclassified	dMMR	MSI-H
P100	55	I	PMS2	c.1970delA:p.Asn657Ilefs	Likely pathogenic	WT	Sporadic	dMMR	MSS
P101	63	III	MLH1	c.380+1G>A	Likely pathogenic	WT	Sporadic	dMMR	MSI-L
P102	65	II	MLH1	c.76C>T:p.Gln26Ter	Pathogenic	MLH1	LS	dMMR	MSI-H
P103	63	III	MLH1	c.380+1G>A	Likely pathogenic	MLH1	LS	dMMR	MSS
P103	63	III	PMS2	c.1970delA:p.Asn657Ilefs	Likely pathogenic	WT	LS	dMMR	MSS
P104	48	II	MLH1	c.199G>A:p.Gly67Arg	Pathogenic	WT	Sporadic	dMMR	MSI-H
P105	61	II	MSH2	c.1699A>T:p.Lys567Ter	Pathogenic	MSH2	LS	dMMR	MSI-H
P106	30	NA	PMS2	c.1970delA:p.Asn657Ilefs	Likely pathogenic	WT	Sporadic	dMMR	MSI-L
P107	71	II	PMS2	c.1970delA:p.Asn657Ilefs	Likely pathogenic	WT	Sporadic	dMMR	MSI-H
P107	71	II	MSH2	c.807dupA:p.L270Tfs*14	Pathogenic	WT	Sporadic	dMMR	MSI-H

Table S2 Continued

Patient	Age (years)	Stage	Gene	Variant	Pathogenicity	Sanger sequencing	LS/sporadic CRCs	MMR_IHC	MSI
P108	39	II	MSH6	c.3487G>T:p.Glu1163Ter; c.3477delC:p.Tyr1159Terfs	Pathogenic	MSH6	LS	dMMR	MSI-H
P109	46	II	MSH6	c.1249_1250delAA:p.Lys417Valfs*5	Pathogenic	Unidentified	Unclassified	dMMR	MSI-H
P110	45	II	PMS2	c.1970delA:p.Asn657Ilefs	Likely pathogenic	WT	Sporadic	dMMR	MSI-H
P111	65	NA	MLH1	c.380+1G>A	Likely pathogenic	MLH1	LS	dMMR	MSI-H
P111	65	NA	PMS2	c.1970delA:p.Asn657Ilefs	Likely pathogenic	WT	LS	dMMR	MSI-H
P112	33	IV	MLH1	c.1975C>T:p.Arg659Ter	Pathogenic	Unidentified	Unclassified	dMMR	MSI-H
P112	33	IV	MLH1	c.1641dupA:p.Y548Ifs*9	Pathogenic	Unidentified	Unclassified	dMMR	MSI-H
P113	44	II	MSH6	c.3013C>T:p.Arg1005Ter	Pathogenic	Unidentified	Unclassified	dMMR	MSS
P114	71	II	PMS2	c.538-2A>G	Likely pathogenic	PMS2	LS	dMMR	MSI-H
P115	45	II	MSH2	c.1599delT:p.R534Vfs*9	Pathogenic	Unidentified	Unclassified	dMMR	MSI-H
P116	48	II	MSH2	c.1699A>T:p.Lys567Ter	Pathogenic	WT	Sporadic	dMMR	MSI-H
P117	42	I	MLH1	c.1989G>A:p.Glu663=	Likely pathogenic	MLH1	LS	dMMR	MSI-H
P118	67	NA	PMS2	c.1970delA:p.Asn657Ilefs	Likely pathogenic	WT	Unclassified	dMMR	MSI-H
P118	67	NA	PMS2	c.444delC:p.Y149Tfs*52	Pathogenic	Unidentified	Unclassified	dMMR	MSI-H
P119	46	I	MSH2	c.1472delA:p.K491Rfs*6	Pathogenic	MSH2	LS	dMMR	MSI-H
P120	56	II	MSH2	c.1699A>T:p.Lys567Ter	Pathogenic	Unidentified	LS	dMMR	MSI-H
P120	56	II	MLH1	c.178C>T:p.Q60*	Pathogenic	MLH1	LS	dMMR	MSI-H
P121	54	NA	MSH2	c.1571G>C:p.Arg524Pro	Likely pathogenic	MSH2	LS	pMMR	MSI-H
P121	54	NA	PMS2	c.1970delA:p.Asn657Ilefs	Likely pathogenic	WT	LS	pMMR	MSI-H
P122	43	NA	MSH2	c.942+3A>T	Pathogenic	MSH2	LS	dMMR	MSI-H
P123	55	NA	PMS2	c.1663C>T:p.Gln555*	Pathogenic	Unidentified	Unclassified	dMMR	MSI-L
P124	48	II	MLH1	c.1852_1854delAAG:p.K618delK	Pathogenic	MLH1	LS	dMMR	MSI-H
P124	48	II	MLH1	c.1896+1_1896+2delinsAC	Pathogenic	Unidentified	LS	dMMR	MSI-H
P125	71	II	PMS2	c.1970delA:p.Asn657Ilefs	Likely pathogenic	WT	Sporadic	dMMR	MSS
P126	46	III	MSH2	c.1699A>T:p.Lys567Ter	Pathogenic	MSH2	LS	dMMR	MSI-H
P127	61	III	PMS2	c.1970delA:p.Asn657Ilefs	Likely pathogenic	WT	LS	dMMR	MSS
P127	61	III	MLH1	c.380+1G>A	Likely pathogenic	MLH1	LS	dMMR	MSS
P127	61	III	MSH6	c.3592_3593insGA:p.A1198Gfs*19	Pathogenic	Unidentified	LS	dMMR	MSS
P128	54	II	MSH6	c.3477delC:p.Tyr1159Terfs	Pathogenic	MSH6	LS	dMMR	MSI-H
P128	54	II	MSH2	c.811_814delTCTG:p.S271Rfs*2	Pathogenic	MSH2	LS	dMMR	MSI-H
P129	38	III	MSH2	c.86delA:p.K29Sfs*35	Pathogenic	Unidentified	Unclassified	dMMR	MSI-H
P130	44	II	MSH2	c.560T>C:p.Leu187Pro	Pathogenic	WT	Sporadic	dMMR	MSS
P131	52	I	MLH1	c.546-2A>C	Pathogenic	MLH1	LS	dMMR	MSI-H
P132	45	I	MLH1	c.230G>A:p.Cys77Tyr	Pathogenic	Unidentified	Unclassified	dMMR	MSI-H

Table S2 Continued

Patient	Age (years)	Stage	Gene	Variant	Pathogenicity	Sanger sequencing	LS/sporadic CRCs	MMR_IHC	MSI
P133	67	III	MLH1	c.1975C>T:p.Arg659Ter	Pathogenic	WT	Sporadic	dMMR	MSS
P133	67	III	PMS2	c.1970delA:p.Asn657Ilefs	Likely pathogenic	WT	Sporadic	dMMR	MSS
P134	56	II	MLH1	c.545G>A:p.Arg182Lys	Pathogenic	MLH1	LS	dMMR	MSI-H
P135	50	III	MSH2	c.1699A>T:p.Lys567Ter	Pathogenic	Unidentified	Unclassified	dMMR	MSI-H
P136	45	II	MSH2	c.2038C>T:p.Arg680Ter	Pathogenic	Unidentified	Unclassified	dMMR	MSS
P137	55	III	MSH6	c.3226C>T:p.Arg1076Cys	Likely pathogenic	MSH6	LS	dMMR	MSI-H
P138	79	NA	PMS2	c.1970delA:p.Asn657Ilefs	Likely pathogenic	WT	Sporadic	dMMR	MSI-H
P139	61	II	MLH1	c.1975C>T:p.Arg659Ter	Pathogenic	WT	Sporadic	dMMR	MSI-H
P139	61	II	PMS2	c.1970delA:p.Asn657Ilefs	Likely pathogenic	WT	Sporadic	dMMR	MSI-H
P140	51	NA	MSH2	c.939delT:p.Q314Rfs*17	Pathogenic	MSH2	LS	dMMR	MSI-H
P141	51	IV	MSH2	c.1699A>T:p.Lys567Ter	Pathogenic	MSH2	LS	dMMR	MSI-H
P142	46	II	MSH6	c.718C>T:p.Arg240Ter	Pathogenic	Unidentified	LS	dMMR	MSI-H
P142	46	II	PMS2	c.1970delA:p.Asn657Ilefs	Likely pathogenic	WT	LS	dMMR	MSI-H
P142	46	II	MSH6	c.3646G>T:p.Gly1216*	Pathogenic	MSH6	LS	dMMR	MSI-H
P143	45	I	MSH2	c.2588_2601del14:p.Y863Sfs*14	Pathogenic	MSH2	LS	dMMR	MSI-H
P144	44	III	MSH2	c.1046C>G:p.Pro349Arg	Pathogenic	MSH2	LS	dMMR	MSI-H
P145	40	NA	PMS2	c.1970delA:p.Asn657Ilefs	Likely pathogenic	WT	Sporadic	pMMR	MSS
P146	52	III	MLH1	c.1732-2A>G	Likely pathogenic	MLH1	LS	dMMR	MSI-H
P147	66	II	PMS2	c.1970delA:p.Asn657Ilefs	Likely pathogenic	WT	Sporadic	dMMR	MSI-H
P148	61	NA	PMS2	c.1970delA:p.Asn657Ilefs	Likely pathogenic	WT	Unclassified	dMMR	MSI-H
P148	61	NA	PMS2	c.1663C>T:p.Gln555*	Pathogenic	Unidentified	Unclassified	dMMR	MSI-H
P149	40	II	MSH2	c.1699A>T:p.Lys567Ter	Pathogenic	MSH2	LS	dMMR	MSI-H
P150	56	II	MSH6	c.3734_3755del22:p.F1245*fs*1	Pathogenic	Unidentified	Unclassified	dMMR	MSI-H
P151	58	II	PMS2	c.1738A>T:p.Lys580Ter	Pathogenic	Unidentified	Unclassified	dMMR	MSI-H
P152	59	II	MSH6	c.956_957insA:p.P320Afs*9; c.1269dupT:p.V424Cfs*11	Pathogenic	Unidentified	LS	dMMR	MSI-H
P152	59	II	MSH2	c.273dupT:p.L92Sfs*8; c.516delA:p. K172Nfs*2	Pathogenic	MSH2	LS	dMMR	MSI-H
P152	59	II	MLH1	c.1509dupC:p.T504Hfs*2	Pathogenic	Unidentified	LS	dMMR	MSI-H
P153	22	II	MSH2	c.1699A>T:p.Lys567Ter	Pathogenic	MSH2	LS	dMMR	MSI-H
P154	54	II	MSH2	c.643_644insG:p.Q215Rfs*17	Pathogenic	MSH2	LS	dMMR	MSI-H

Table S3 Demographic and clinicopathological features of LS, dMMR/MSI-H sporadic CRCs, and non-dMMR/MSI-H sporadic CRCs

Parameters	LS, n = 88	Sporadic_dMMR/ MSI-H, n = 292	P value	LS, n = 88	Sporadic_non-dMMR/ MSI-H, n = 248	P value	Sporadic_dMMR/ MSI-H, n = 292	Sporadic_non-dMMR/ MSI-H, n = 248	P value
	n (%)	n (%)		n (%)	n (%)		n (%)	n (%)	
Gender			0.48			0.54			0.89
Male	54 (61.4)	165 (57.1)		54 (61.4)	143 (57.7)		165 (57.1)	143 (57.7)	
Female	34 (38.6)	124 (42.9)		34 (38.6)	105 (42.3)		124 (42.9)	105 (42.3)	
Age			0.016			0.016			0.93
<55	53 (60.2)	107 (36.6)		53 (60.2)	90 (36.3)		107 (36.6)	90 (36.3)	
≥55	35 (39.8)	185 (63.4)		35 (39.8)	158 (63.7)		185 (63.4)	158 (63.7)	
Clinical stage			0.59			<0.001			<0.001
I	11 (13.6)	33 (12.0)		11 (13.6)	11 (5.0)		33 (12.0)	11 (5.0)	
II	53 (65.4)	163 (59.5)		53 (65.4)	66 (30.3)		163 (59.5)	66 (30.3)	
III	15 (18.5)	71 (25.9)		15 (18.5)	117 (53.7)		71 (25.9)	117 (53.7)	
IV	2 (2.5)	7 (2.6)		2 (2.5)	24 (11.0)		7 (2.6)	24 (11.0)	
Location			0.12			<0.001			<0.001
Left	26 (32.5)	105 (42.3)		26 (32.5)	190 (79.5)		105 (42.3)	190 (79.5)	
Right	54 (67.5)	143 (57.7)		54 (67.5)	49 (20.5)		143 (57.7)	49 (20.5)	
Differentiation			0.73			<0.001			<0.001
Low	21 (33.3)	63 (29.9)		21 (33.3)	30 (13.0)		63 (29.9)	30 (13.0)	
Medium	38 (60.3)	138 (65.4)		38 (60.3)	193 (83.5)		138 (65.4)	193 (83.5)	
High	4 (6.3)	10 (4.7)		4 (6.3)	8 (3.5)		10 (4.7)	8 (3.5)	
Mucinous			0.1			<0.001			<0.001
No	10 (33.3)	75 (49.7)		10 (33.3)	207 (83.5)		75 (49.7)	207 (83.5)	
Yes	20 (66.7)	76 (50.3)		20 (66.7)	41 (16.5)		76 (50.3)	41 (16.5)	

dMMR, deficient mismatch repair; MSI-H, high microsatellite instability, LS: Lynch syndrome.

Table S4 Demographic and clinicopathological features of LS subgroups with different germline variant genes and sporadic CRCs

	MLH1_LS, *n* = 33	Sporadic, *n* = 540	*P* value	MSH2_LS, *n* = 39	Sporadic, *n* = 540	*P* value	MSH6_LS, *n* = 13	Sporadic, *n* = 540	*P* value	PMS2_LS, *n* = 6	Sporadic, *n* = 540	*P* value
Parameters	*n* (%)	*n* (%)		*n* (%)	*n* (%)		*n* (%)	*n* (%)		*n* (%)	*n* (%)	
Gender			0.08			0.67			0.8			0.97
Male	24 (72.7)	308 (57.4)		21 (53.8)	308 (57.4)		7 (53.8)	308 (57.4)		4 (66.7)	308 (57.4)	
Female	9 (27.3)	229 (42.6)		18 (46.2)	229 (42.6)		6 (46.2)	229 (42.6)		2 (33.3)	229 (42.6)	
Age (yr)			< 0.001			< 0.001			0.32			0.15
<55	23 (69.7)	197 (36.5)		25 (64.1)	197 (36.5)		7 (53.8)	197 (36.5)		0 (0)	197 (36.5)	
≥55	10 (30.3)	343 (63.5)		14 (35.9)	343 (63.5)		6 (46.2)	343 (63.5)		6 (100)	343 (63.5)	
Clinical stage			0.067			0.032			0.47			0.067
I	6 (20.0)	44 (8.9)		3 (8.6)	44 (8.9)		1 (7.7)	44 (8.9)		2 (33.3)	44 (8.9)	
II	17 (56.7)	229 (46.5)		25 (71.4)	229 (46.5)		9 (69.2)	229 (46.5)		4 (66.7)	229 (46.5)	
III	6 (20.0)	188 (38.2)		6 (17.1)	188 (38.2)		3 (23.1)	188 (38.2)		0 (0)	188 (38.2)	
IV	1 (3.3)	31 (6.3)		1 (2.9)	31 (6.3)		0 (0)	31 (6.3)		0 (0)	31 (6.3)	
Location			0.005			< 0.001			0.29			0.92
Left	9 (33.3)	295 (60.6)		9 (24.3)	295 (60.6)		6 (46.2)	295 (60.6)		3 (50)	295 (60.6)	
Right	18 (66.7)	192 (39.4)		28 (75.7)	192 (39.4)		7 (53.8)	192 (39.4)		3 (50)	192 (39.4)	
Differentiation			0.17			0.29			0.51			0.011
Low	7 (36.8)	93 (21.0)		10 (33.3)	93 (21.0)		2 (20.0)	93 (21.0)		2 (33.3)	93 (21.0)	
Medium	11 (57.9)	331 (74.9)		19 (63.3)	331 (74.9)		7 (70.0)	331 (74.9)		2 (33.3)	331 (74.9)	
High	1 (5.3)	18 (4.1)		1 (3.3)	18 (4.1)		1 (10.0)	18 (4.1)		2 (33.3)	18 (4.1)	
Mucinous			0.29			0.006			0.016			0.088
No	5 (50.0)	282 (70.7)		4 (30.8)	282 (70.7)		1 (16.7)	282 (70.7)		0 (0)	282 (70.7)	
Yes	5 (50.0)	117 (29.3)		9 (69.2)	117 (29.3)		5 (83.3)	117 (29.3)		2 (100)	117 (29.3)	
MSI			< 0.001			< 0.001			0.008			0.012
MSI-H	27 (81.8)	221 (40.9)		38 (97.4)	221 (40.9)		11 (84.6)	221 (40.9)		6 (100)	221 (40.9)	
MSI-L	1 (3.0)	11 (2.0)		0 (0)	11 (2.0)		0 (0)	11 (2.0)		0 (0)	11 (2.0)	
MSS	5 (15.2)	308 (57.0)		1 (2.6)	308 (57.0)		2 (15.4)	308 (57.0)		0 (0)	308 (57.0)	
MMR-IHC			< 0.001			< 0.001			< 0.001			0.065
dMMR	33 (100)	291 (53.9)		38 (97.4)	291 (53.9)		13 (100)	291 (53.9)		6 (100)	291 (53.9)	
pMMR	0 (0)	249 (46.1)		1 (2.6)	249 (46.1)		0 (0)	249 (46.1)		0 (0)	249 (46.1)	

MMR, mismatch repair; dMMR, deficient MMR; pMMR, proficient MMR; MSI, microsatellite instability; MSI-H, MSI-high; MSI-L, MSI-low; MSS, microsatellite stability, LS: Lynch syndrome.

Table S5 Demographic and clinicopathological features of LS CRCs in subgroups with different germline variant genes

	MLH1_LS, n = 33	Non_MLH1_LS, n = 55	P value	MSH2_LS, n = 39	Non_MSH2_LS, n = 49	P value	MSH6, n = 13	Non_MSH6_LS, n = 75	P value	PMS2_LS, n = 6	Non_PMS2_LS, n = 82	P value
Parameters	n (%)	n (%)		n (%)	n (%)		n (%)	n (%)		n (%)	n (%)	
Gender			0.09			0.2			0.55			1
Male	24 (72.7)	30 (54.5)		21 (53.8)	33 (67.3)		7 (53.8)	47 (62.7)		4 (66.7)	50 (61.0)	
Female	9 (27.3)	25 (45.5)		18 (46.2)	16 (32.7)		6 (46.2)	28 (37.3)		2 (33.3)	32 (39.0)	
Age (yr)			0.16			0.51			0.61			0.007
<55	23 (69.7)	30 (54.5)		25 (64.1)	28 (57.1)		7 (53.8)	46 (61.3)		0 (0)	53 (64.6)	
≥55	10 (30.3)	25 (45.5)		14 (35.9)	21 (42.9)		6 (46.2)	29 (38.7)		6 (100)	29 (35.4)	
Clinical stage			0.49			0.69			0.87			0.31
I	6 (20.0)	5 (9.8)		3 (8.6)	8 (17.4)		1 (7.7)	10 (14.7)		2 (33.3)	9 (12.0)	
II	17 (56.7)	36 (70.6)		25 (71.4)	28 (60.9)		9 (69.2)	44 (64.7)		4 (66.7)	49 (65.3)	
III	6 (20.0)	9 (17.6)		6 (17.1)	9 (19.6)		3 (23.1)	12 (17.6)		0 (0)	15 (20.0)	
IV	1 (3.3)	1 (2.0)		1 (2.9)	1 (2.2)		0 (0)	2 (2.9)		0 (0)	2 (2.7)	
Location			0.91			0.15			0.25			0.62
Left	9 (33.3)	17 (32.1)		9 (24.3)	17 (39.5)		6 (46.2)	20 (29.9)		3 (0.5)	23 (31.1)	
Right	18 (66.7)	36 (67.9)		28 (75.7)	26 (60.5)		7 (53.8)	47 (70.1)		3 (0.5)	51 (68.9)	
Differentiation			0.92			0.64			0.59			0.015
Low	7 (36.8)	14 (31.8)		10 (33.3)	11 (33.3)		2 (20.0)	19 (35.8)		2 (33.3)	19 (33.3)	
Medium	11 (57.9)	27 (61.4)		19 (63.3)	19 (57.6)		7 (70.0)	31 (58.5)		2 (33.3)	36 (63.2)	
High	1 (5.3)	3 (6.8)		1 (3.3)	3 (9.1)		1 (10.0)	3 (5.7)		2 (33.3)	2 (3.5)	
Mucinous			0.34			1			0.63			0.54
No	5 (50.0)	5 (25.0)		4 (30.8)	6 (35.3)		1 (16.7)	9 (37.5)		0 (0)	10 (35.7)	
Yes	5 (50.0)	15 (75.0)		9 (69.2)	11 (64.7)		5 (83.3)	15 (62.5)		2 (100)	18 (64.3)	
MSI			0.061			0.17			0.38			1
MSI-H	27 (81.8)	53 (96.4)		38 (97.4)	42 (85.7)		11 (84.6)	69 (92.0)		6 (100)	74 (90.2)	
MSI-L	1 (3.0)	0 (0)		0 (0)	1 (2.0)		0 (0)	1 (1.3)		0 (0)	1 (1.2)	
MSS	5 (15.2)	2 (3.6)		1 (2.6)	6 (12.2)		2 (15.4)	5 (6.7)		0 (0)	7 (8.5)	

Table S5 Continued

Parameters	MLH1_LS, n = 33	Non_MLH1_LS, n = 55	*P* value	MSH2_LS, n = 39	Non_MSH2_LS, n = 49	*P* value	MSH6, n = 13	Non_MSH6_LS, n = 75	*P* value	PMS2_LS, n = 6	Non_PMS2_LS, n = 82	*P* value
	n (%)	*n* (%)		*n* (%)	*n* (%)		*n* (%)	*n* (%)		*n* (%)	*n* (%)	
MMR-IHC			1			0.44			1			1
dMMR	33 (100)	54 (98.2)		38 (97.4)	49 (100)		13 (100)	74 (98.7)		6 (100)	81 (98.8)	
pMMR	0 (0)	1 (1.8)		1 (2.6)	0 (0)		0 (0)	1 (1.3)		0 (0)	1 (1.2)	

MMR, mismatch repair; dMMR, deficient MMR; pMMR, proficient MMR; MSI, microsatellite instability; MSI-H, MSI-high; MSI-L, MSI-low; MSS, microsatellite stability, LS: Lynch syndrome.

Table S6 Performance of MMR IHC and MSI testing for LS pre-screening

	Sensitivity (%)	Specificity (%)	PPV (%)	NPV (%)
dMMR	98.9	46.1	23.0	99.6
MSI-H	90.9	59.1	26.6	97.6
MSI-H/L	92.0	57.0	25.9	97.8

MMR, mismatch repair; MSI, microsatellite instability; PPV, positive predictive value; NPV, negative predictive value.

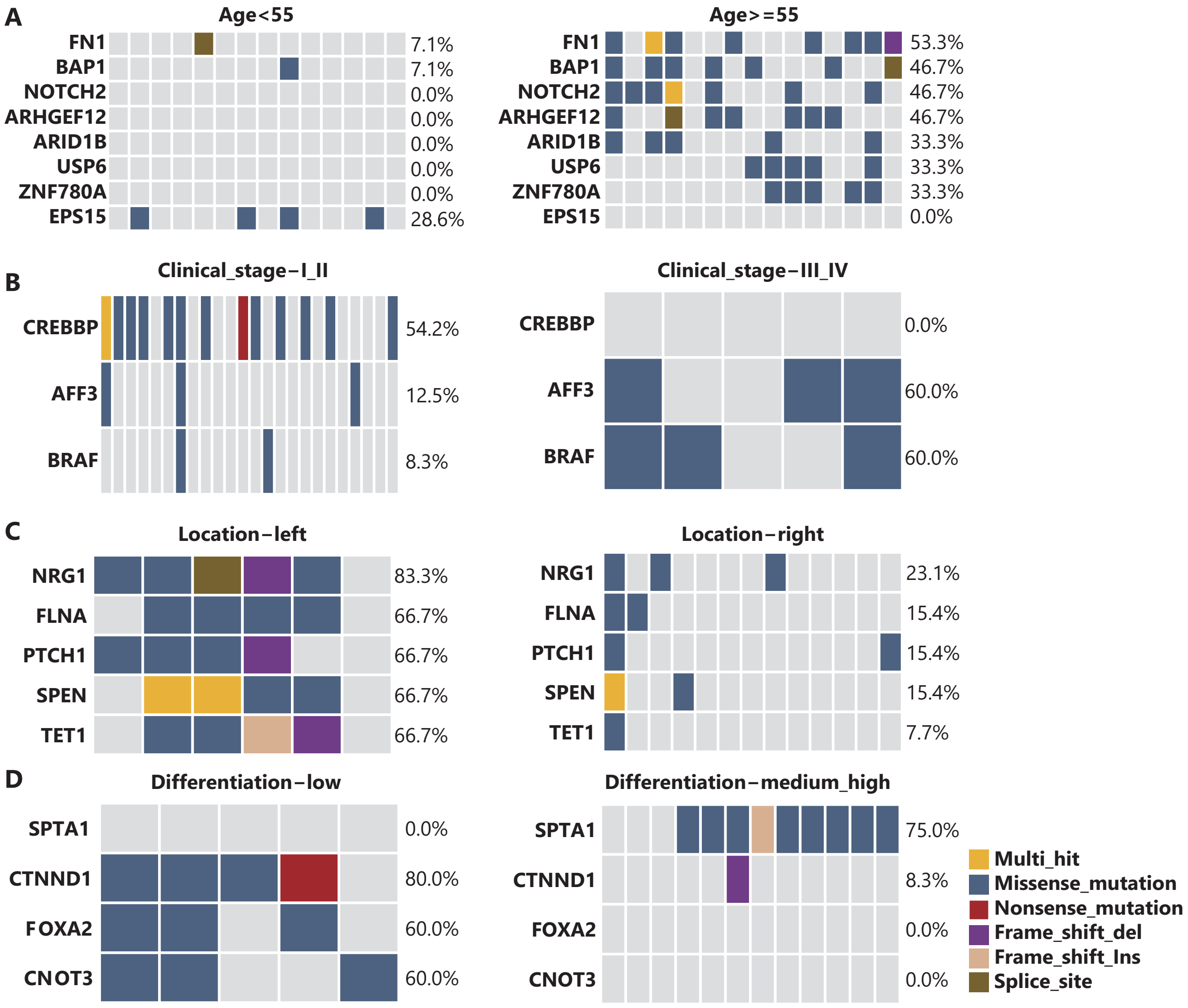

Figure S1 Comparison of gene variant frequencies in groups with different clinical features. (A–D) Cancer associated genes with significantly different variant frequencies in groups with different ages, clinical stages, tumor locations, and degrees of differentiation. Each row represents a gene, each column represents a sample, and colors represent types of variants.

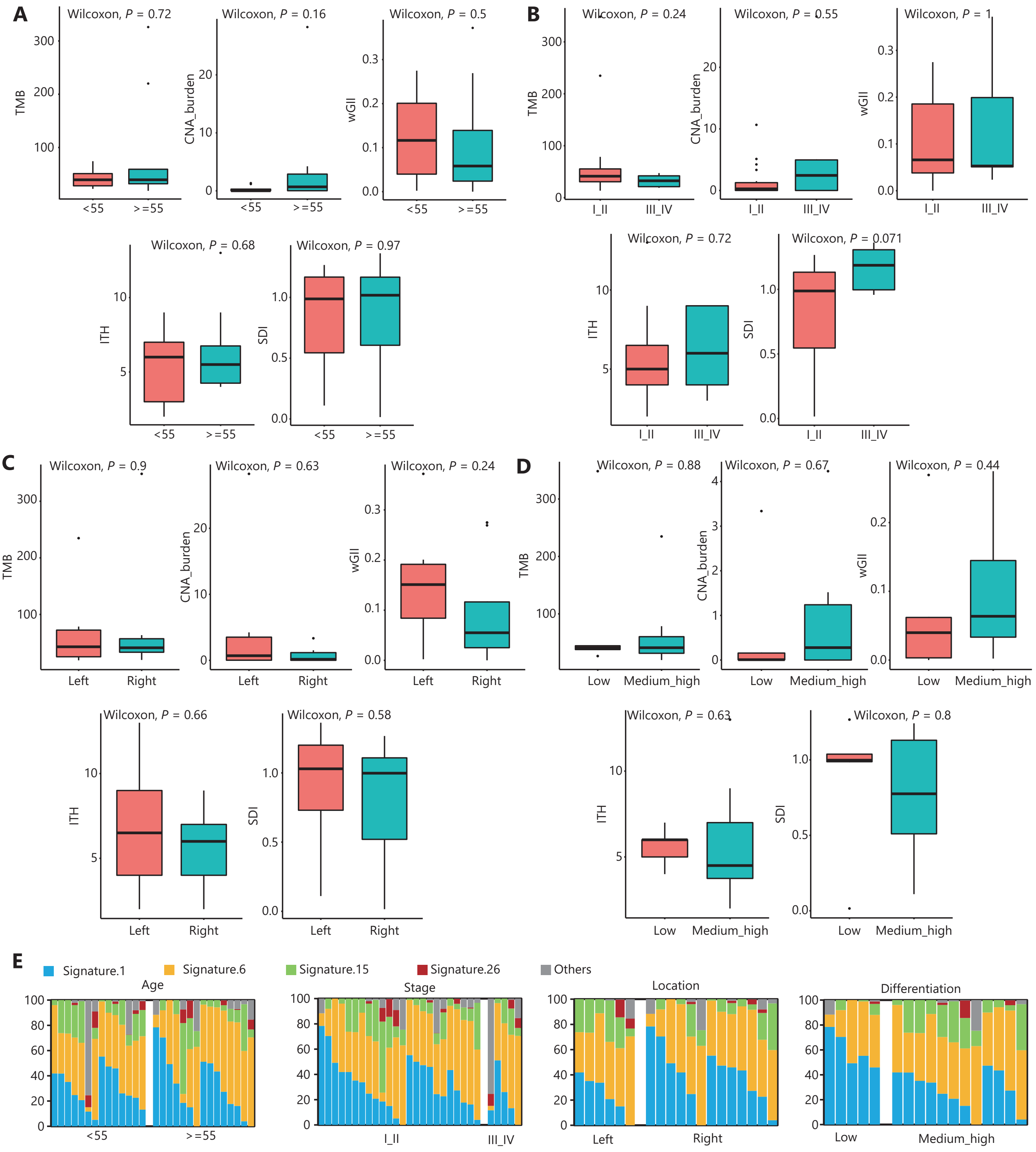

Figure S2 Comparison of genetic features in groups with different clinical features. (A) TMB, CNV burden, wGII, ITH index, and SDI in groups with different ages. (B) TMB, CNV burden, wGII, ITH index, and SDI in groups with different clinical stages. (C) TMB, CNV burden, wGII, ITH index, and SDI in groups with different tumor locations. (D) TMB, CNV burden, wGII, ITH index, and SDI in groups with different degrees of differentiation. (E) Mutational signatures in groups with different ages, clinical stages, tumor locations, and degrees of differentiation.
